# The effects of stochastic resonance electrical stimulation and neoprene sleeve on knee proprioception

**DOI:** 10.1186/1749-799X-4-3

**Published:** 2009-02-02

**Authors:** Amber T Collins, J Troy Blackburn, Chris W Olcott, Douglas R Dirschl, Paul S Weinhold

**Affiliations:** 1 Department of Biomedical Engineering, University of North Carolina, Chapel Hill, NC, USA; 2 Department of Orthopaedics, University of North Carolina, Chapel Hill, NC, USA; 3 Department of Exercise and Sports Science, University of North Carolina, Chapel Hill, NC, USA; 4 Program in Human Movement Science, University of North Carolina, Chapel Hill, NC, USA

## Abstract

**Background:**

A variety of knee injuries and pathologies may cause a deficit in knee proprioception which may increase the risk of reinjury or the progression of disease. Stochastic resonance stimulation is a new therapy which has potential benefits for improving proprioceptive function. The objective of this study was to determine if stochastic resonance (SR) stimulation applied with a neoprene sleeve could improve knee proprioception relative to a no-stimulation/no-sleeve condition (control) or a sleeve alone condition in the normal, healthy knee. We hypothesized that SR stimulation when applied with a sleeve would enhance proprioception relative to the control and sleeve alone conditions.

**Methods:**

Using a cross-over within subject design, twenty-four healthy subjects were tested under four combinations of conditions: electrical stimulation/sleeve, no stimulation/sleeve, no stimulation/no sleeve, and stimulation/no sleeve. Joint position sense (proprioception) was measured as the absolute mean difference between a target knee joint angle and the knee angle reproduced by the subject. Testing was conducted during both partial-weight bearing (PWB) and non-weight bearing (NWB) tasks. Differences in joint position sense between the conditions were evaluated by repeated-measures analysis of variance testing.

**Results:**

Joint position sense error during the stimulation/sleeve condition (2.48° ± 1.32°) was found to be more accurate (P < 0.05) relative to the control condition (3.35° ± 1.63°) in the PWB task. No difference in joint position sense error was found between stimulation/sleeve and sleeve alone conditions for the PWB task. Joint position sense error was not found to differ between any of the conditions for the NWB task.

**Conclusion:**

These results suggest that SR electrical stimulation when combined with a neoprene sleeve is an effective modality for enhancement of joint proprioception in the PWB knee. We believe these results suggest the need for further study of the potential of SR stimulation to correct proprioceptive deficits in a clinical population with knee injury/pathology or in subjects at risk of injury because of a proprioceptive deficit.

## Background

Proprioception is the conscious and unconscious awareness of body limb position and movement. Proprioception is traditionally measured by joint position sense (JPS) or joint movement sense (joint kinesthesia) [1,2]. The degree of weight bearing (WB) is an important aspect of measuring JPS. Studies [2,3] have evaluated the influence of weight bearing on JPS and have found JPS to be significantly more accurate in a WB task compared to a nonWB task (NWB) [3]. Thus, the WB status may provide different proprioceptive information due to different contributions of mechanoreceptors being stimulated [3,4].

Knee proprioception deficits have a role in several clinical conditions or injuries. Knee proprioceptive deficits are known to occur after anterior cruciate ligament tears [5], and proprioceptive training has been investigated as a means of preventing these injuries [6]. Knee proprioception deficits are exacerbated in the elderly [7,8], and this is believed to be a factor contributing to the risk of falls in this population. Furthermore, knee proprioceptive deficits have been shown to be greater in subjects with knee osteoarthritis (OA) than in elderly age-matched controls [7,9,10], and it is believed these deficits may contribute to the progression of osteoarthritis [11].

The clinical conditions associated with knee proprioception deficits have stimulated interest in methods by which proprioception may be improved. Several studies have demonstrated an improvement in knee proprioception with the use of a neoprene knee sleeve or brace in the NWB knee [12-14] with no improvement in the WB knee [14]. An additional therapy for improving proprioception is exercise. Laskowski et al. has described the use of balance training and kinetic chain exercises to improve proprioception [4]. A new therapy which has potential benefits for improving proprioceptive function is the use of subthreshold electrical stimulation via a phenomenon known as stochastic resonance (SR). SR is a phenomenon in which the response of nonlinear systems (e.g. somatosensory) to weak input signals can be optimized in the presence of a specific low level of noise (mechanical or electrical). The net result of the SR stimulation is heightened somatosensory sensitivity. SR effects were initially shown to increase the sensitivity of cutaneous [15] and muscle spindle receptor systems [16]. More recently, Gravelle et al. [17] investigated the effects of SR stimulation applied at the knee and found a reduction in postural sway in elderly subjects. Enhancement of somatosensory function through the use of SR has also been tested in subject populations with diabetes, stroke patients, and functional ankle instability [8,18].

The present study was designed to evaluate proprioception in the normal knee under various combinations of neoprene sleeve and SR electrical stimulation conditions. The objective of this study was to determine whether random subthreshold SR electrical stimulation applied in combination with a sleeve to the normal knee would improve proprioception as measured by JPS during both a NWB and partial WB (PWB) task. Our primary hypothesis was that proprioception would be more accurate during the sleeve/stimulation condition compared to the no sleeve/no stimulation control condition. Our secondary hypothesis was that proprioception would improve with the application of the SR stimulation and sleeve combination beyond the improvement seen with the sleeve alone.

## Methods

### Subjects

Prior to participation, all subjects read and signed an informed consent form which had previously been approved by the Institutional Review Board. Twenty-four (12 males, 12 females) healthy, physically active subjects between 18 and 35 years of age were recruited. Subject descriptive statistics are presented in Table 1. Subjects were excluded if they had a history of functional instability of the knee joint, previous knee surgery, current knee injury or functional instability, or any known neurological conditions which could prevent the subject from sensing motion or feeling pain. Additionally, subjects were excluded if they had a history of cardiac arrhythmia, a history of gait or postural disorders, seizures, diabetes, fainting, peripheral neuropathy, stroke, motion sickness, or if they were required to have a cardiac pacemaker or drug delivery pump.

**Table 1:** Subject demographics (Mean ± SD, N = 24)

	**Female (N = 12)**	**Male (N = 12)**	**Group (N = 24)**
**Age (yr)**	25.08 ± 3.99	24.58 ± 3.53	24.96 ± 3.72
**Mass (kg)**	61.42 ± 7.70	81.31 ± 13.00	68.91 ± 20.51
**Height (in.)**	64.75 ± 1.86	70.25 ± 1.60	67.65 ± 3.27
**BMI**	22.68 ± 2.43	25.52 ± 4.01	24.17 ± 3.61

### Study Design

JPS was evaluated during both a PWB and a NWB task under the following four conditions: no electrical stimulation/no sleeve (NE/NS), no electrical stimulation/sleeve (NE/S), electrical stimulation/no sleeve (E/NS), electrical stimulation/sleeve (E/S). Testing was performed on the subject's dominant knee, with dominance defined as the limb used to kick a ball for maximal distance. JPS was measured as the ability to actively reproduce a target knee flexion angle. Similar studies have used target angles of knee flexion in the range of 20 to 40 degrees because this range simulates stance phase flexion during walking, and is reported to be strongly associated with proprioceptive feedback during normal walking [9]. The target angle used in this study was 30 degrees. To prevent any memorization effect of the target angle, a "dummy" 60 degree target angle was also incorporated into the PWB testing sequence and a "dummy" 50 degree target angle was incorporated into the NWB testing sequence. The dummy angle stages used the electrical stimulation and sleeve condition of the previous stage of the test sequence (Table 2). One sequence was assigned to each subject for his/her first task (PWB or NWB). The second task was then completed with the sequence number shown in Table 2. Data at the dummy angle was not analyzed. A counterbalanced design was used in developing the sequence that the testing conditions were introduced to each subject.

**Table 2:** Listing of the 24 total test sequences that incorporate the dummy target angles for the PWB and NWB tasks

Sex	**PWB/NWB**	**1st Task Sequence**	**A**	**B**	**C**	**D**	**E**	**F**	**2nd Task Sequence**
M	PWB	1	+E/-S	**60 deg**	-E/-S	**60 deg**	-E/+S	+E/+S	23
M	PWB	2	-E/+S	**60 deg**	+E/+S	**60 deg**	-E/-S	+E/-S	21
M	PWB	3	-E/-S	**60 deg**	+E/-S	**60 deg**	+E/+S	-E/+S	22
M	PWB	4	+E/+S	**60 deg**	-E/+S	**60 deg**	+E/-S	-E/-S	24
M	PWB	5	+E/-S	**60 deg**	-E/-S	+E/+S	**60 deg**	-E/+S	19
M	PWB	6	+E/+S	**60 deg**	-E/+S	-E/-S	**60 deg**	+E/-S	20
M	NWB	7	-E/+S	**50 deg**	+E/+S	+E/-S	**50 deg**	-E/-S	17
M	NWB	8	-E/-S	**50 deg**	+E/-S	-E/+S	**50 deg**	+E/+S	18
M	NWB	9	+E/-S	-E/-S	**50 deg**	-E/+S	**50 deg**	+E/+S	14
M	NWB	10	-E/+S	+E/+S	**50 deg**	-E/-S	**50 deg**	+E/-S	13
M	NWB	11	-E/-S	+E/-S	**50 deg**	+E/+S	**50 deg**	-E/+S	16
M	NWB	12	+E/+S	-E/+S	**50 deg**	+E/-S	**50 deg**	-E/-S	15
F	PWB	13	+E/-S	**60 deg**	-E/-S	-E/+S	**60 deg**	+E/+S	10
F	PWB	14	-E/+S	**60 deg**	+E/+S	-E/-S	**60 deg**	+E/-S	9
F	PWB	15	-E/-S	**60 deg**	+E/-S	+E/+S	**60 deg**	-E/+S	12
F	PWB	16	+E/+S	**60 deg**	-E/+S	+E/-S	**60 deg**	-E/-S	11
F	PWB	17	+E/-S	**60 deg**	-E/-S	**60 deg**	+E/+S	-E/+S	7
F	PWB	18	+E/+S	**60 deg**	-E/+S	**60 deg**	-E/-S	+E/-S	8
F	NWB	19	-E/+S	**50 deg**	+E/+S	**50 deg**	+E/-S	-E/-S	5
F	NWB	20	-E/-S	**50 deg**	+E/-S	**50 deg**	-E/+S	+E/+S	6
F	NWB	21	+E/-S	-E/-S	**50 deg**	+E/+S	**50 deg**	-E/+S	2
F	NWB	22	+E/+S	-E/+S	**50 deg**	-E/-S	**50 deg**	+E/-S	3
F	NWB	23	-E/+S	+E/+S	**50 deg**	+E/-S	**50 deg**	-E/-S	1
F	NWB	24	-E/-S	+E/-S	**50 deg**	-E/+S	**50 deg**	+E/+S	4

A-F represent different stages of the test sequence.

The orders of weight-bearing status (PWB vs. NWB), stimulation/sleeve conditions, and testing angle (target vs. "dummy") were introduced via a counterbalanced design.

### Equipment

Electrical stimulation was applied with an electrical stimulator system (Afferent Corporation, Providence, RI) by way of two pairs of self-adhesive surface electrodes (ValuTrode Model CFF125, Axelgaard, Fallbrook, CA). The stimulation system consisted of one computer with Labview software, a multifunction DAQ card, two analog stimulus isolation boxes, two error isolation boxes, and two pairs of surface electrodes. Electrode pairs (stimulator and ground) were placed approximately 2 cm above and below the joint line, respectively. Once the electrodes were placed, they remained in position throughout all testing conditions. Stimulation consisted of a 50 μA Gaussian white noise signal (zero mean, s.d. = 0.05 mA, 0–1000 Hz bandwidth) and was controlled via Labview software. This stimulation was confirmed to be below the subject's threshold of detection for each electrode pair and has been previously applied at the knee to improve postural sway in elderly subjects [17].

**Figure 1:**
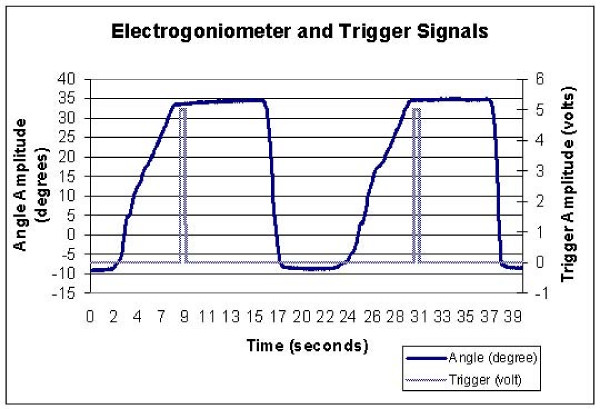
**Joint angle and electronic trigger signals that were acquired during a testing trial**.

JPS was also tested while wearing a neoprene knee sleeve. Each subject wore one of four sleeve sizes (Small, Medium, Large, Extra Large) based on a secure, but not uncomfortable fit as reported by the subject. A calibrated electrogoniometer was aligned with the sagittal plane knee joint axis of rotation and strapped to the lateral side of the dominant knee. The electrogoniometer was interfaced with a PC data acquisition board that acquired the knee flexion angle in real-time (100 Hz) during the testing which gave an electronic readout of the knee angle with accuracy to less than 0.5°. During both the PWB and the NWB testing sequences, the subject was instructed to momentarily depress an electronic trigger when they arrived at the target angle during the learning task and also when they felt they had reproduced the target angle during the reproduction task. The electronic trigger provided a time stamp for when the target angle was achieved (Figure 1).

### Procedures

**Figure 2:**
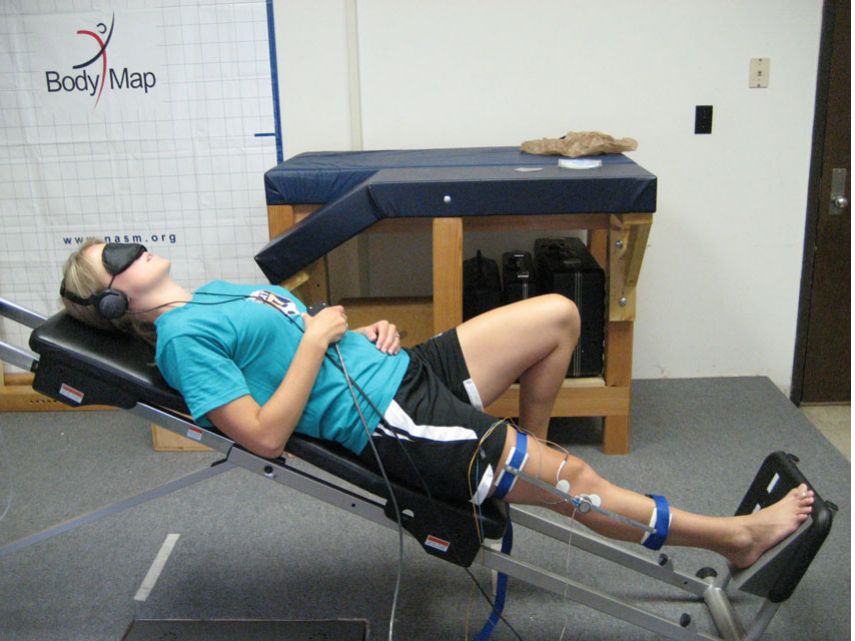
**Partial Weight Bearing (PWB) setup simulating single leg stance**.

Subjects wore a blindfold and headphones during all trials in order to minimize visual and auditory cues. White noise was played on the headphones only during the angle reproduction portion of each trial to ensure the subject could hear instructions provided by the investigator during the initial presentation of the target angle. During the PWB task, subjects were instructed to lie on a reclined sliding platform (20°) that was relatively frictionless with the test limb extended and foot resting on a heel wedge (Figure 2). Given the angle of the platform with respect to vertical, the ground reaction force imparted to the subject was approximately 34% WB. The heel wedge was introduced to decrease passive tension generated in the triceps surae muscle group. The nontest limb was flexed at the hip and knee with the foot resting on the sliding platform. The subject began the test with the knee at the starting angle of 0° flexion, and was instructed to slowly flex the test limb until told to stop by the investigator (i.e. when the target knee flexion angle was attained). Once stopped, the subject depressed an electronic trigger and held this position for at least 5 seconds. The subject then returned to the starting position. After a rest period of at least 5 seconds the investigator began the headphone noise and tapped the subject on the nontest limb to instruct them to begin flexing the test limb in order to reproduce the target angle. Once the subject reached what he/she perceived to be the target angle, he/she depressed the electronic trigger and held this position for at least 5 seconds. The subject then returned to the starting position, thus completing a single trial.

**Figure 3:**
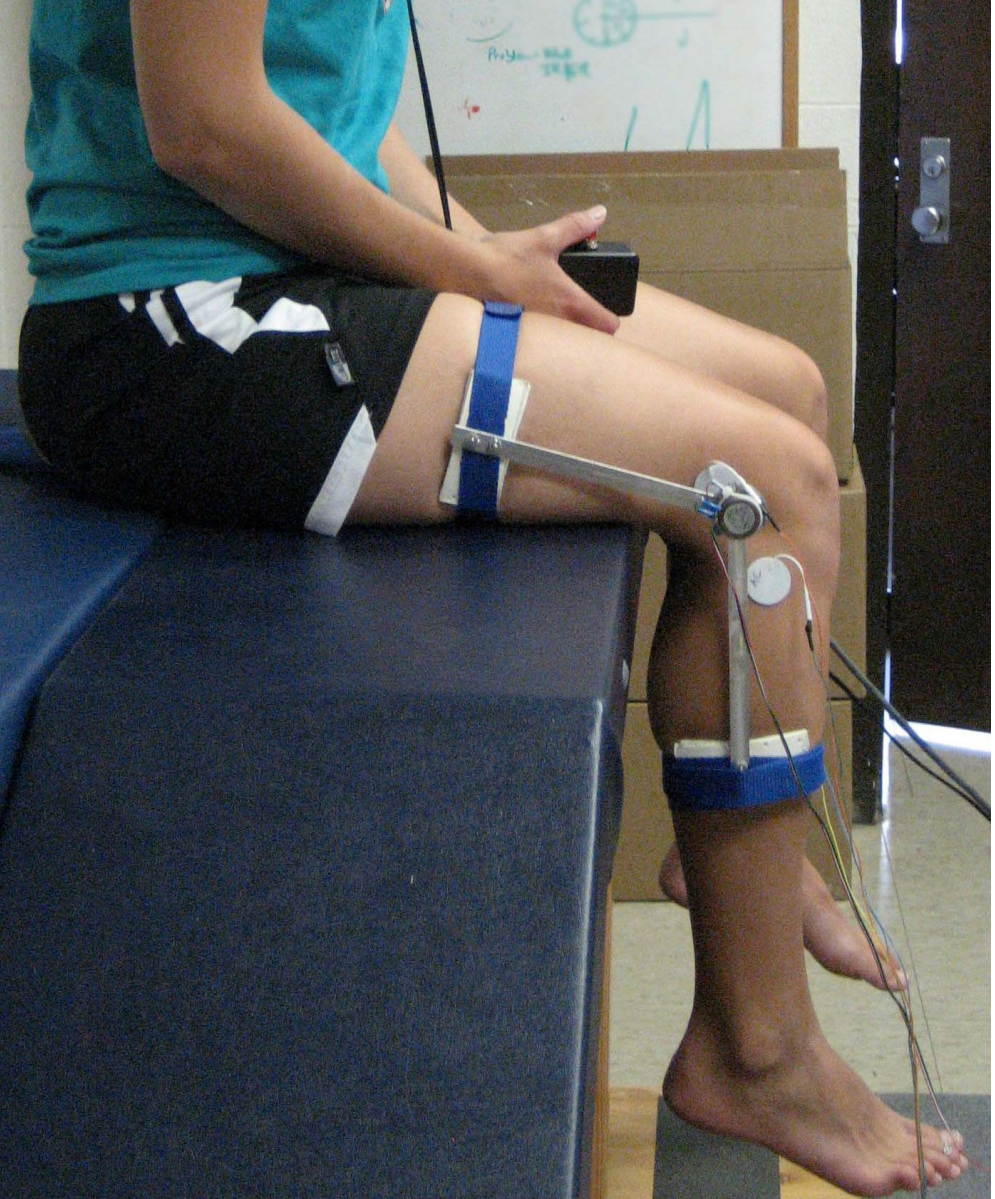
**Non Weight Bearing (NWB) setup simulating the swing phase of walking**.

In the NWB task, subjects were positioned on a bench seated upright with their legs freely hanging over the edge and the popliteal space a few centimeters off the bench edge (Figure 3). The subject began with the knee resting at 70°–80° degrees flexion, and the test limb was passively extended by the investigator until the target angle was reached. Subsequently, the subject depressed the electronic trigger and held the position for at least 5 seconds. Following a 5 second rest, the subject actively repositioned their limb to the target angle similar to the PWB task. Three trials were completed for each of the four conditions in both the PWB and NWB tasks. Other studies have shown that this method of determining joint position sense is reliable and accurate [2,19]. JPS was defined as the absolute value of the difference between the target and reposition angle (identified as the knee angle during the respective time periods in each task during which the electronic trigger was depressed) for each of the three trials and averaged. This "absolute error" was used in the data analysis.

### Statistical Analysis

A priori statistical power analysis determined that testing 24 subjects would be sufficient to yield an angle reproduction improvement of 30% with a standard deviation of the absolute error of angle reproduction of 50% of the mean. The standard deviation and percentage improvement were conservative estimates based on the results of previously reported NWB and PWB studies [9,13,14]. A power of 0.8 and significance level of 0.05 were used.

The NWB and PWB data were not normally distributed and a Friedman repeated measures analysis of variance on ranks was performed to determine overall significance. Frequency distributions of all four conditions were examined and they appeared normal and were found to conform to skewness and kurtosis values for normality. A one-way (4 conditions) repeated-measures analysis of variance (ANOVA) followed by Holm-Sidak posthoc tests were conducted to determine differences in the measured variable with the four conditions for each task. Two-way (stimulus and sleeve status) repeated measures ANOVA was also conducted.

A linear regression analysis was performed to determine if improvements in proprioception across the test conditions were dependent on the absolute error of the control condition (NE/NS). A greater improvement would be expected in subjects who produced a larger mean absolute error in the control condition. Change scores were calculated as the difference in the absolute error between the control condition and each stimulus/sleeve combination condition. Linear regression was used to evaluate the relationships between these change scores of absolute error (dependent variables) and the control condition absolute error (independent variable).

To determine whether the application of electrical stimulation had any lasting effects on the errors of the control condition, a one-way ANOVA was conducted to examine if the control condition error changed as its relative position in the task sequence changed. An unpaired t-test was used to assess the influence of gender on the absolute error.

## Results

**Figure 4:**
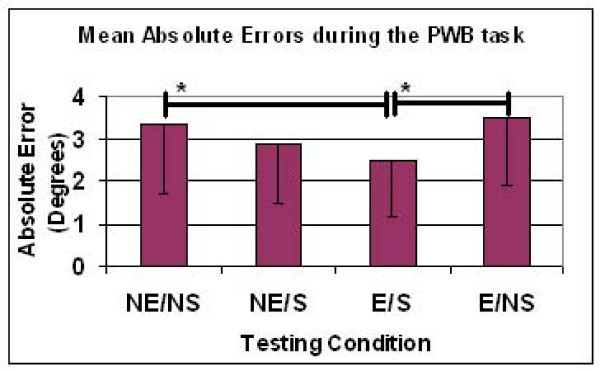
**Absolute error for the four conditions for the partial weight bearing (PWB) joint position sense testing**. * indicates significant difference (P < 0.05) between conditions at ends of horizontal bar.

The results of the one-way ANOVA for the PWB task revealed a significant effect of the testing condition. Specifically, the mean absolute error of the stimulation/sleeve condition (E/S: 2.48° ± 1.32°) was significantly decreased (P < 0.05) relative to the control condition (NE/NS: 3.35° ± 1.63°). However, the mean absolute error of the E/S condition did not differ from the sleeve alone condition (NE/S: 2.87° ± 1.41°), and the NE/S condition was not found to differ from the control condition. Finally, the stimulus alone condition (E/NS: 3.48° ± 1.58°) was not found to differ from the control (NE/NS) or sleeve alone conditions (NE/S). The results for each of the test conditions for the PWB task are summarized in Figure 4 and Table 3. The two-way ANOVA revealed a significant (P = 0.014) main effect of the sleeve, but no main effect of the stimulus.

**Table 3:** Mean absolute errors (in degrees) and Standard Deviations (SD) for all four conditions in the PWB task

**PWB Task**	**Mean (SD)**	*Mean difference (95% CI of difference)	**Mean difference (95% CI of difference)
**No Electrical Stimulation/No Sleeve (NE/NS)**	3.35† (1.63)	N/A	0.48 (-0.31 to 1.27)

**No Electrical Stimulation/Sleeve (NE/S)**	2.87 (1.41)	-0.48 (-1.27 to 0.31)	N/A

**Electrical Stimulation/Sleeve (E/S)**	2.48†‡ (1.32)	-0.86 (-1.68 to -0.050)	-.39 (-1.12 to 0.34)

**Electrical Stimulation/No Sleeve (E/NS)**	3.48‡ (1.58)	0.13 (-0.63 to 0.89)	0.61 (-0.17 to 1.40)

Significant differences were found between the E/S and NE/NS conditions (†) and between the E/S and E/NS conditions (‡). The mean differences (95% confidence interval) between each condition and the control (NE/NS)* as well as the mean differences between each condition and the sleeve only (NE/S)** condition are shown.

For the NWB task no significant differences were detected between conditions for the one-way ANOVA. The mean absolute error for each of the conditions for the NWB task were the following: NE:NS (5.86° ± 3.80°), NE:S (4.96° ± 3.52 °), E:S (5.69° ± 3.73°), and E:NS (5.89° ± 3.74°). These results are summarized in Table 4. The results of the two-way ANOVA for the NWB task revealed no significant main effects due to the stimulation or sleeve.

**Table 4:** Mean absolute errors (in degrees) and Standard Deviations (SD) for all four conditions in the NWB task

**NWB Task**	**Mean (SD)**	*Mean difference (95% CI of difference)	**Mean difference (95% CI of difference)
**No Electrical Stimulation/No Sleeve (NE/NS)**	5.86 (3.80)	N/A	0.90 (-0.12 to 1.92)

**No Electrical Stimulation/Sleeve (NE/S)**	4.96 (3.52)	-0.90 (-1.92 to 0.12)	N/A

**Electrical Stimulation/Sleeve (E/S)**	5.69 (3.73)	-0.16 (-1.24 to 0.91)	0.73 (-0.17 to 1.64)

**Electrical Stimulation/No Sleeve (E/NS)**	5.89 (3.74)	0.04 (-0.73 to 0.80)	0.94 (-0.08 to 1.95)

No significant differences were detected between any of the four conditions. The mean differences (95% confidence interval) between each condition and the control (NE/NS)* as well as the mean differences between each condition and the sleeve only (NE/S)** condition are shown.

**Figure 5:**
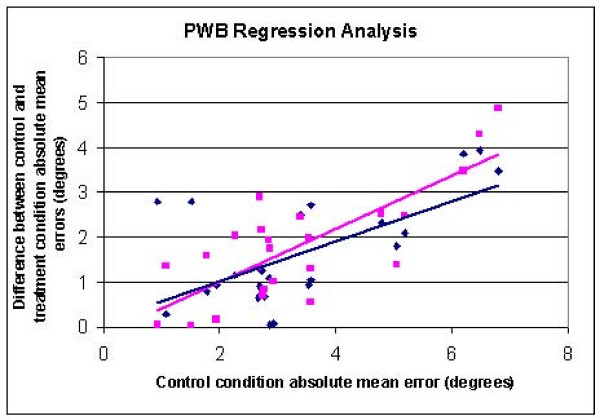
**Regression analysis of the partial weight bearing (PWB) data for the improvement in joint position sense error (in degrees) with condition versus the control error (diamond = NE/NS-NE/S, square = NE/NS-E/S)**.

The regression analysis revealed a significant relationship between the improvement in mean absolute error for the E/S (R = 0.618, P = 0.001) and NE/S (R = 0.780, P < 0.001) conditions and the mean absolute error of the control condition for the PWB task (Figure 5). The regression equations for these relationships were the following: E/S improvement (y = 0.5938x - 0.1874), NE/S improvement (y = 0.4453x + 0.1293). For the NWB task the relationship between the E/S improvement and the mean absolute error of the control condition was significant (R = 0.54, P = 0.006), but relationships with the other test conditions were not found to be significant. No gender effects were present for either the PWB or NWB data. The relative position of the control condition in the test sequence was not found to influence the control condition error.

## Discussion

In support of our primary hypothesis, a significant improvement in JPS relative to the control condition was found when SR stimulation was applied with a neoprene sleeve in normal subjects during a PWB task. The importance of the combined SR stimulation and sleeve condition is that the clinical application of SR stimulation would undoubtedly be applied by electrodes incorporated into some form of sleeve, brace, or garment. Thus, the current findings show some promise for the clinical application of SR stimulation to enhance knee proprioception. The observation that the improvement in proprioception occurred in the PWB knee is also important in that it suggests the potential for this condition to improve joint positioning in a more highly loaded knee when the knee is typically at greater risk of injury. Our findings correspond with a previous study of SR electrical stimulation of the knee which has shown that postural sway can be reduced during single-legged stance in older adults[17]. Somatosensory information is critical to balance control and this previous study indirectly suggested the potential of SR stimulation to enhance the sensitivity of the somatosensory system and improve knee proprioception.

For the PWB task, our findings that JPS was no different with SR in combination with the sleeve when compared with the sleeve alone were contrary to our hypothesis. However, our mean comparison testing also showed the JPS of the sleeve alone condition and the SR stimulation alone condition were each not different from the control condition in the PWB task. These results highlight the importance of the sleeve in enabling enhancement of knee proprioception with SR stimulation. Past studies have shown that sleeves or braces can enhance knee proprioception in the NWB knee [12,13]. However similar to our findings, a past study has been unable to demonstrate a significant improvement in knee proprioception with the presence of a sleeve/brace in the WB or loaded knee [14]. It is unclear why the SR stimulation alone was unable to improve JPS, but it may be that the presence of the sleeve increased coupling at the skin-electrode interface during limb movement.

For the NWB task we were unable to detect any improvements in JPS with any of the 4 conditions, and thus were unable to provide support for our hypothesis under NWB conditions. It is unclear if the lack of an effect with the SR stimulation/sleeve condition in the NWB task was a result of us being unable to detect this effect, or if there was truly no such effect. The absence of an effect of SR stimulation with the sleeve in the NWB condition could be because the mechanoreceptors contributing proprioceptive input in the NWB limb were not specifically targeted by the SR stimulation. In addition, a lack of an effect could also suggest that joint tissues may have to be prestressed for the mechanoreceptors residing in them to be more responsive to the SR stimulus. Additionally, since our standard deviation values for the NWB task were greater than the estimated 50% that was set in our priori power analysis, it is possible that a type II error may have occurred. Similar to past studies, our data showed a pattern for the sleeve alone to enhance knee proprioception, however this did not prove to be statistically significant in our study. Birmingham et al. [14] demonstrated a 1.2 degree decrease in absolute mean error when a sleeve was added during a sitting open kinetic chain exercise in healthy young adults. Herrington et al. [13] demonstrated a 0.6 degree difference in mean absolute error between the no sleeve and sleeve conditions for subjects seated in a NWB position. Specific to this study, we saw a 0.90 degree difference in mean absolute error when the sleeve was added compared with the control condition. When comparing the absolute error values of the NWB task to the PWB task it can be observed that the errors are larger for the NWB. This pattern agrees with past studies that have shown JPS to be more accurate during WB tasks than for NWB tasks [2,3]. Investigators have suggested that the improved JPS present in the WB limb is likely due to increased proprioceptive information being available [2,3,14]. This augmented proprioceptive information may be coming from adjacent joints or because of enhanced stimulation of mechanoreceptors of the joint of interest when tissues are loaded.

The small magnitude of improvement in JPS with the SR stimulation/sleeve condition in the PWB task may prompt some to question the clinical significance of this effect. While it is difficult to define what magnitude of improvement in error is clinically significant, studies examining the influence of proprioceptive training on knee function provide some indication that the observed difference may prove clinically significant. Tsauo et al. 2008 [20] and Lin et al. 2007 [21] each conducted randomized clinical trials to evaluate the effect of proprioceptive training exercises on knee proprioception and self-reported knee function in patients with knee osteoarthritis. Both studies found improvements in the absolute error of JPS testing of approximately 2 degrees with training. Both studies also reported a significant improvement in self-reported function (WOMAC index) with training that occurred in parallel with the improvement of proprioceptive acuity. These studies are suggestive that minor improvements in proprioception acuity can cause significant changes in function.

An important consideration in interpreting the results of this study is that the improvements seen with the treatment conditions may have been limited by utilizing young, healthy adults. Our regression analyses indicated that larger proprioceptive improvements occurred in individuals with larger initial errors for the control condition. This observation leads us to believe that enhancements in knee proprioception with the SR stimulation/sleeve condition may be greater in a clinical population that has a knee proprioceptive deficit. There are several clinical populations with a knee proprioceptive deficit that could be the focus of future studies with SR stimulation. Knee proprioception is known to be impaired with aging, knee osteoarthritis (OA), and ACL injury [7,10,22]. The effect of the SR stimulation/sleeve condition could be examined in each of these populations to determine if improvements in proprioception are greater than those observed in healthy subjects.

While we believe this study is important with valid results, it was not without limitations. Lasting effects of the stimulation may have been a limitation as they could have affected results in subsequent conditions; however the counterbalanced design of our study likely minimized such an effect, and our analysis indicated no effect of the relative location of the control condition within the task sequence. The fitting of the neoprene knee sleeve may have also been a limitation. The sleeve was fitted for each subject based on comfort. Hassan et al. [19] tested JPS in OA subjects while the subjects wore one of two types of bandages, with one fitting more loosely than the other. They found a significant improvement in proprioception acuity with the looser bandage, but no improvement with the standard fit bandage. Specific to our study, we felt the neoprene sleeve was fit securely enough to provide the necessary support, although the degree of cutaneous mechanoreceptor stimulation may have varied across subjects. The use of a single target angle may have been a limitation as well. It is possible that despite the use of "dummy angles" incorporated throughout the testing sequence, a memorization effect may have remained. Testing joint position sense with only 3 repetitions may have also been a limitation as some studies suggest there should be at least 5 repetitions before stable data can be assumed [23,24]. Selfe et al. evaluated the effect of increasing the number of test trials in the assessment of knee joint position sense by measuring the progression of means and standard deviations as the trial number increased [23]. Their goal was to determine the point at which the mean and standard deviation could be considered to have stabilized and this was set as the point where the standard deviation changed by less than 5 percent of the cumulative mean. The progression of the means and standard deviations were calculated specific to this study and we found the change in standard deviation was within 5 percent of the cumulative mean after only three trials in all conditions of the NWB task and in all but one condition of the PWB task (E:NS). The stimulation alone (E:NS) condition was not statistically significant. Additionally, we believed conducting 5 repetitions would have extended an already lengthy testing session likely causing the subjects to lose focus during the testing. Similar studies testing knee proprioception have used 3 repetitions [2,19]. A final limitation may be that the level of stimulus may not have been high enough to elicit activation of the specific mechanoreceptors required for proprioceptive acuity.

## Conclusion

Overall, our objective was to determine whether subthreshold SR electrical stimulation applied in combination with a sleeve to the normal knee would improve proprioception. It was found that SR stimulation when applied with a neoprene knee sleeve could improve proprioception in the PWB knee. In contrast, no such effect was detected in the NWB knee. The results of this study show promise toward developing an effective therapy for treating knee proprioceptive deficits. As the subjects in the current investigation were healthy, young adults with normal proprioception, the improvements in proprioception with SR stimulation may have been limited due to a ''ceiling effect''. We feel more research is necessary to determine the effect of SR electrical stimulation on JPS in clinical populations with proprioceptive deficits such as knee OA patients.

## Competing interests

The authors declare that they have no competing interests.

## Authors' contributions

AC performed all subject testing, data collection, data analysis, and drafted the manuscript. TB assisted with study design and critically revised the manuscript. CO assisted with study design. DD helped with study conception, procured funding, and critically revised the manuscript. PW conceived and designed the study and helped to draft the manuscript. All authors read and approved the final manuscript.
